# Trends of Advanced Chronic Liver Disease Among 17,711 Persons in Mongolia During Years 2015–2023

**DOI:** 10.1111/jvh.70129

**Published:** 2026-01-06

**Authors:** Habiba Kamal, Ganbolor Jargalsaikhan, Sanjaasuren Enkhtaivan, Daniel Bruce, Karin Lindahl, Bekhbold Dashtseren, Tuvshinjargal Ulziibadrakh, Munguntsetseg Batkhuu, Purevjargal Bat‐Ulzii, Sumiya Byambabaatar, Soo Aleman, Naranjargal B. Dashdorj

**Affiliations:** ^1^ Department of Medicine Huddinge Karolinska Institute Stockholm Sweden; ^2^ The Department of Infectious Diseases Karolinska University Hospital Stockholm Sweden; ^3^ The Liver Centre Ulaanbaatar Mongolia; ^4^ ONOM Foundation Ulaanbaatar Mongolia; ^5^ Cytel Statistical Consultancy Stockholm Sweden

**Keywords:** cirrhosis, HBV, HCV, HDV, mongolia

## Abstract

Chronic liver diseases cause a significant burden in Asia. This study aims to characterise the pattern and factors associated with advanced chronic liver disease (aCLD) in a large cohort from Mongolia, which has the highest rate of liver cancer globally. This is a cross‐sectional analysis of HBsAg tested adults (≥ 18 years) with available liver stiffness measurement (LSM) and/or platelet count at initial visit at a hepatology centre, Ulaanbaatar, Mongolia during 2015–2023. Definition of aCLD was LSM ≥ 12.5 kPa or platelet count < 150*10^9^ cells/L. The annual percentage change (APC) of the incidence rates and factors associated with aCLD were assessed via logistic regression models. Out of 17,711 persons identified, 2517 (14.2%) had aCLD at initial visit, with a mean age (±SD) of 50.1 (12.0) years, 23.6% below 40 years old and 53.2% were female. The prevalence of HBsAg+, anti‐HDV+, HDV RNA+, and anti‐HCV+ was 61.6%, 78.6%, 64.6% and 57.9% in aCLD, respectively. Among aCLD, three‐fourths had an intermediate to high 5‐year risk of hepatocellular carcinoma (HCC) with 25.0%, and 17.1% having previously received anti‐HBV and anti‐HCV therapies, respectively. The overall rate of aCLD declined (APC −7.8%), mainly due to decline in anti‐HCV+ cases (APC −15.0%), while it significantly increased for anti‐HDV+ over study period (APC 3.3%). In a large cohort of attendees at a hepatology centre in Mongolia, the prevalence of aCLD declined associated with decreasing HCV infection, while chronic hepatitis D constituted the majority of increasing cases. A minority received therapy, with most patients showing an intermediate to high risk of liver cancer. More efforts are needed to improve linkage to care and access to therapy, especially in middle‐aged individuals at higher risk of liver disease progression.

**Abbreviations**
aCLD: advanced chronic liver disease
APC: annual percentage change
HBV: hepatitis B virus
HCC: hepatocellular carcinoma
HCV: hepatitis C virus
HDV: hepatitis D virus
LSM: liver stiffness measurement
MASLD: metabolic dysfunction‐associated steatotic liver disease
MRF: metabolic risk factor
PAGE‐B score: platelets age gender risk score

## 1 Introduction

Chronic liver disease causes a significant health burden in Asia [1]. In 2019, nearly 50% and 75% of global deaths related to advanced chronic liver disease (aCLD) and hepatocellular carcinoma (HCC), respectively, occurred in the Asia‐pacific region [1]. Nonetheless, aCLD, including cirrhosis is a cause of disability‐adjusted life‐years, highly impacting people aged 25–50 years [2]. Patients with chronic liver disease are often underdiagnosed, with 30%–50% having already developed cirrhosis at diagnosis, missing timely interventions [2, 3].

Studies have associated this late diagnosis with multiple factors such as age, sex, ethnicity, health infrastructure, comorbidities, socioeconomic and environmental disparities [1, 4]. While the majority of aCLD and HCC in Asia are attributed to chronic infection with hepatitis B, C and D viruses (HBV, HCV, HDV), metabolic‐associated and alcohol‐related liver disease are increasing possibly due to urbanisation and economic affluence [5, 6]. Despite these figures, a comprehensive epidemiological profile of aCLD from Central Asian populations has not been reported. Nevertheless, knowledge about recent trends regarding the prevalence of viral hepatitis and, specifically, the predictors for aCLD is still limited. Therefore, this study aims to analyse age‐ and sex‐specific trends and causes of aCLD in a large cohort of 17,711 individuals diagnosed with viral hepatitis attending a hepatology clinic in Ulaanbaatar, Mongolia over the past decade [7]. Mongolia has the highest mortality rate of HCC in the world, with a heavy burden of advanced liver disease [8]. Such information is important to guide health policy decisions and to prioritise interventions with the greatest impact to reduce liver disease morbidity and mortality, which is especially relevant to low‐ and middle‐income settings [5].

## 2 Materials and Methods

### 2.1 Study Design, Population and Setting

This was an observational, cross‐sectional analysis of adults (≥ 18 years) attending the Liver Centre in Ulaanbaatar, Mongolia between January 2015 and December 2023, with at least one HBsAg test. This centre provides outpatient care for persons referred or self‐seeking care for symptoms of liver diseases. An overall 51,113 individuals were identified from the centre database. Persons with missing birthdates, sex, or age < 18 years were excluded (Figure S1 study flowchart).

### 2.2 Study Participants

Study participants were sub grouped based on liver stiffness measurements (LSM) ≥ 12.5 kPa or platelet count less than 150 cells × 10 [9] /litre into patients with or without record of aCLD [9]. Further subgrouping by virological parameters included: HBsAg+ and HBsAg‐, identification of individuals with HBV mono‐infection: HBsAg+/anti‐HDV‐; those with HBV/HDV co‐infection: HBsAg+/anti‐HDV+; those with chronic hepatitis D (CHD): HBsAg+/anti‐HDV+ and HDV RNA+ ≥ 50 IU/mL; those with resolved or active HCV infection: anti‐HCV+; and those with chronic hepatitis C (CHC): HCV RNA+ ≥ 10 IU/mL.

### 2.3 Baseline Variables and Definition

The date of the first HBsAg test was considered the baseline date for this study. Demographic characteristics such as sex, birth date, body mass index (BMI) and date of the first HBsAg test were ascertained from the centre database. BMI was calculated as weight in kilogram/ (height in meters) [2] and a cutoff of ≥ 23.0 kg/m^2^ was considered to indicate overweight or obesity [10]. Other laboratory parameters such as alanine aminotransferase (ALT), aspartate aminotransferase (AST), gamma glutamyl transferase (GGT), platelet count, blood glucose and other biochemical indices were also retrieved. This, while virological data included quantitative HBsAg, anti‐HBs, HBV DNA, anti‐HCV antibody, HCV RNA, HBeAg status, anti‐HDV antibody and quantitative HDV RNA.

Liver stiffness measurements (LSM) and CAP ranging from 2.0 to 75.0 kPa, and 100 to 400 dB per meter (dB/m), respectively were assessed by transient elastography. LSM was considered reliable when the success rate of measurements was ≥ 90% and the interquartile range (IQR) was ≤ 30% according to local routine [9]. LSM cutoffs suggestive of liver fibrosis stages were defined as no or minimal fibrosis (F0‐F1) for LSM < 7.5 kPa; ≥ 7.5‐ < 9.0 kPa as F2; ≥ 9.0‐ < 12.5 kPa as F3 and ≥ 12.5 as F4 [9]. For patients missing LSM values, a platelet count < 150 × 10^9^ cells/L was defined as a surrogate of advanced fibrosis/cirrhosis [11]. Liver cancer diagnosis and dates were ascertained by reviewing patients’ journals.

Elevated GGT was defined as GGT ≥ 55 IU/L in men, and ≥ 38 IU/L in women. Patients who received interferon‐based, nucleo(s)tides analogues (NAs) and novel anti‐HDV bulevirtide at the time of HBsAg test were identified. Therapies were initiated at physicians’ discretion, and usually renewed monthly at the liver centre. A metabolic risk factor (MRF) was defined as the presence of at least one of the following: overweight/obesity, diabetes mellitus (either a documented diagnosis or fasting serum glucose ≥ 7.0 mmol/L on two occasions), dyslipidemia, hypertension and/or diagnosis of fatty liver disease [12]. We categorised patients according to the PAGE‐B score which is based on age, sex and platelets count to assess the longitudinal 5‐year risk of HCC primarily, primarily aimed at chronic hepatitis B patients under NAs therapy [13]. Co‐medication data (e.g., statins, beta‐blockers, aspirin) were not available in the database.

### 2.4 Laboratory Tests

Biochemical parameters and complete blood count were analysed using an automated BX‐3010 chemistry analyser (Sysmex Co. Ltd., Japan) and XN‐550 analyser (Sysmex Co. Ltd., Japan), respectively. HBsAg, anti‐HCV and anti‐HIV were qualitatively tested by rapid diagnostic test (CTK Co. Ltd., USA), while anti‐HDV were tested using ELISA (Wantai Co. Ltd., PRC). Quantitative HBsAg was estimated by HISCL‐5000 fully automated chemiluminescence analyser (Sysmex Co. Ltd., Japan) and for HBeAg status (CLIA, Sysmex, Japan, lower level of detection (LOD) of 1 COI). HBV DNA, HCV RNA and HDV RNA were quantified using real time reverse transcriptase polymerase chain reaction with LOD of 10 IU/mL (GeneXpert, Cepheid, USA), 10 IU/mL (GeneXpert, Cepheid, USA) and 50 IU/mL (Bioactiva diagnostica, Germany; Boi‐Rad, USA), respectively. Laboratory and virological parameters were considered within 90 days of the first HBsAg test and for platelets and LSM within 365 days.

### 2.5 Ethical Aspects

This study is a collaboration between the Liver Centre, Mongolia, and the research group at the Department of Infectious Diseases, Karolinska Institute, Sweden.

All data were anonymized and statistical analyses were conducted at Karolinska institute. Ethical approval was granted by the local ethics review boards at Mongolia and Sweden and the study conformed to the 1975 Helsinki declaration. Individual patient consent was waived as the study involved anonymized register data. The study was funded by a grant from ALF, Region Stockholm, Sweden.

### 2.6 Study Outcome

The primary outcome was to characterise the temporal changes in the prevalence of aCLD over 2015–2023 at the time of their initial HBsAg test in the records. The secondary outcome was to assess the temporal patterns of aCLD by sex, stratified by age 40 years and by chronic hepatitis B, C and D.

### 2.7 Statistical Analyses

Continuous variables were presented as means (standard deviation, SD) or medians (IQR, interquartile range). Student‐t and Mann–Whitney U tests were used for the comparison of continuous variables. Categorical variables were presented as frequencies (numbers) and proportions and were compared using the Chi‐Square test or Fisher's exact test, as appropriate. The crude incidence rate (CIR) was computed as the number of persons with aCLD divided by the total number of attendees per visit year presented as a proportion. To assess the temporal changes in the prevalence of aCLD, we calculated the annual percentage change (APC) using a negative binomial regression model with 95% confidence interval (CI). Overdispersion was assessed using the Pearson *x* ^2^ statistic and supported the use of a negative binomial model over a standard Poisson model. The number of aCLD cases per visit year was modelled as the dependent variable, with year (centred at 2015) as a continuous predictor and the log of the total number of patients as an offset to account for varying population sizes. Rates and APCs stratified by sex, age group (< 40, ≥ 40 years), HBV monoinfection, anti‐HDV+, anti‐HCV+ and MRF were evaluated to delineate the risk of aCLD in different populations. Univariable and multivariable logistic regression analysis yielding odds ratio (OR with 95% CI) were used to identify baseline factors associated with aCLD. Statistical significance was defined at *p* < 0.05, analyses were performed using R (version 4.4.1; R Foundation for Statistical Computing, Vienna, Austria) and IBM SPSS Statistics (version 27.0; IBM Corp., Armonk, NY, USA).

## 3 Results

### 3.1 Study Cohort

From 2015 to 2023, we identified 51,113 consecutive individuals who underwent at least one HBsAg assessment at the Liver Centre, Mongolia. A cohort of 48,522 (95%) met the eligibility criteria, from which we selected 17,711 patients with available LSM and platelet count as presented in Figure S1.

### 3.2 Characteristics of Persons Diagnosed With aCLD at Initial HBsAg Test and Subgrouped by Sex

Of 17,711 persons with available LSM and/or platelets count, 2517 (14.2%) had LSM ≥ 12.5 and/or platelet count < 150*10^9^ cells/L. Females constituted 53.2%, at a significantly older age of 53.6 (11.2) years versus 46.1 (11.6) years in males (*p* < 0.001) (Table 1). The prevalence of patients younger than 40 years of age with aCLD was 23.6%, constituting 14.1% in females and 34.3% in males (*p* < 0.001). (Table 1, Table S1).

**TABLE 1:** Baseline characteristics of 17,711 individuals subgrouped by record of advanced chronic liver disease (aCLD) at initial visit during 2015–2023.

Variables	Available	All	No record of aCLD	aCLD	*p*
**Number**		17,711	15,194	2517	
Age at HBsAg test, years, mean (sd)	17,711	45.2 (12.7)	44.4 (12.6)	50.1 (12.0)	< 0.001
Age < 40 years of age	17,711	6988 (39.5)	6395 (42.1)	593 (23.6)	< 0.001
**Sex**	17,711				0.005
Female		9875 (55.8)	8537 (56.2)	1338 (53.2)	
Male		7836 (44.2)	6657 (43.8)	1179 (46.8)	
BMI, kg/m^2^, median (IQR)	5847	26.6 (23.7, 29.8)	26.6 (23.7, 29.8)	26.7 (23.9, 30.7)	0.11
ALT, IU/L, median (IQR)	17,118	41.9 (24.0, 78.0)	38.7 (22.7, 71.0)	68.6 (39.9, 117.0)	< 0.001
AST, IU/L, median (IQR)	17,117	32.4 (21.3, 55.1)	29.6 (20.3, 47.8)	62.0 (38.6, 98.2)	< 0.001
GGT, IU/L, median (IQR)	13,893	36.0 (21.5, 66.5)	33.3 (20.5, 59.0)	63.9 (35.2, 119.9)	< 0.001
Platelets count, 10*9 cells/L, median (IQR)	17,287	222.0 (178.0, 268.0)	234.0 (197.0, 275.0)	123.0 (94.0, 142.0)	< 0.001
FBG level, mmol/L, median (IQR)	14,614	5.1 (4.7, 5.6)	5.1 (4.7, 5.5)	5.1 (4.6, 5.8)	0.002
Diabetes mellitus	14,614	1239 (8.5)	962 (7.7)	277 (13.5)	< 0.001
**Virological Parameters**					
No record of viral hepatitis B, C or D	17,711	3856 (21.8)	3708 (24.4)	148 (5.9)	< 0.001
HBsAg+	17,711	9158 (51.7)	7608 (50.1)	1550 (61.6)	< 0.001
HBeAg positive	2147	827 (38.5)	692 (37.6)	135 (43.8)	0.038
Any anti‐HDV or HDV RNA test record	17,711	9418 (53.2)	7840 (51.6)	1578 (62.7)	< 0.001
HBsAg+/anti‐HDV—	9418	1983 (21.1)	1893 (24.1)	90 (5.7)	< 0.001
Anti‐HDV+ or HDV RNA+	9418	5982 (63.5)	4741 (60.5)	1241 (78.6)	< 0.001
HDV RNA+	5982	3665 (61.3)	2863 (60.4)	802 (64.6)	< 0.001
Any anti‐HCV or HCV RNA test record	17,711	12,167 (68.7)	10,434 (68.7)	1733 (68.9)	0.91
Anti‐HCV+ or HCV RNA+	12,167	5476 (45.0)	4472 (42.9)	1004 (57.9)	< 0.001
HCV RNA+	5476	4195 (76.6)	3408 (76.2)	787 (78.4)	< 0.001
qHBsAg log_10_, IU/mL, median (IQR)	7988	3.6 (2.9, 4.0)	3.6 (2.9, 3.9)	3.7 (3.2, 4.0)	< 0.001
qHBV DNA log_10_, IU/mL, median (IQR)	6792	5.2 (3.8, 6.1)	2.7 (1.7, 3.6)	2.3 (1.3, 3.2)	< 0.001
qHDV RNA log_10_, IU/mL, median (IQR)	3760	2.6 (1.6,3.6)	5.2 (3.7, 6.1)	5.4 (4.3, 6.1)	< 0.001
qHCV RNA log_10_, IU/mL, median (IQR)	4195	5.8 (4.3, 6.4)	5.9 (4.3, 6.4)	5.8 (3.9, 6.3)	0.06
LSM, kPa, median (IQR)	3622	7.6 (5.5, 11.3)	6.8 (5.2 8.8)	17.6 (14.4, 24.8)	< 0.001
Previous NAs	9158	1645 (18.0)	1257 (16.5)	388 (25.0)	< 0.001
Previous DAAs	2388	379 (15.9)	305 (15.6)	74 (17.1)	0.4
MRF	17,711	7019 (39.6)	6070 (39.9)	949 (37.7)	0.033
Decompensation baseline	17,711	127 (0.7)		127 (5.0)	na
HCC baseline	17,711	111 (0.6)	65 (0.4)	46 (1.8)	< 0.001

*Note:* Parameters are provided as *n* (%) unless stated otherwise.

Abbreviations: ALT = alanine aminotransferase, AST = aspartate aminotransferase, BMI = body mass index, DAAS = direct acting antivirals, FBG = fasting blood glucose, GGT = gamma glutamyl transferase, HCC = hepatocellular carcinoma, IQR = interquartile range, LSM = liver stiffness measurement, MRF = metabolic risk factor, NAs = nucleotide analogues, na = not applicable, sd = standard deviation, ULN = upper limit of normal.

The overall prevalence of HBsAg+, anti‐HDV+ and anti‐HCV+ was 61.6%, 78.6% and 57.9%, respectively, while patients with any MRF constituted 37.7% and 5.9% had no record of chronic viral hepatitis. (Figure S2 shows the distribution of these parameters by visit year).

In Table S1, males had a more prevalent HBsAg+ (68.2% vs. 55.8%, *p* < 0.001), more HBeAg+ (52.8% vs. 34.2%, *p* < 0.001) and more anti‐HDV+ (83.0% vs. 74.5%, *p* < 0.001) compared to females. In contrast, females had a more prevalent detectable HDV RNA+ (68.2% vs. 61.3%, *p* = 0.012), more anti‐HCV+ (66.8% vs. 47.4%, *p* < 0.001) and more HCV RNA+ (80.5% vs. 74.8%) compared to males. At the initial HBsAg test, 46 (1.8%) patients were diagnosed with HCC, with male patients constituting 56.5% (26/46) of them. Despite having a similar BMI to females, males had higher blood glucose levels, more diabetes (16.2% vs. 11.2%, *p* < 0.001) and a more prevalent MRF (41.4% vs. 34.5%, *p* < 0.001). Overall, 388 (25.0%) of those with HBV received prior NAs therapy, with a similar distribution between male and female patients (*p* = 0.6).

Figure S3 shows the distribution of PAGE‐B risk score in HBsAg+ with aCLD; where male patients ≥ 40‐years of age had a 7.4% prevalence of high‐risk, while most females fell into intermediate and low risk groups (*p* < 0.001). Similarly, most male patients younger than 40 fell into the intermediate risk group at 86.9% versus 4.7% in young female peers, (*p* < 0.001).

### 3.3 Characteristics of Persons With aCLD by Age

Male patients constituted the majority in the < 40 age group (68.1%), with a higher prevalence of overall HBsAg+, HBV monoinfection, anti‐HDV+ and HDV RNA+ in the younger group (all *p* < 0.001), in contrast to the higher prevalence of anti‐HCV+ and HCV RNA+ in the older age group (*p* < 0.001). MRF was more prevalent in the older group (39.2% vs. 32.7%, *p* = 0.004), as well as more HCC diagnosis at baseline (*p* < 0.001). Of note, exposure to NAs was similar between both age groups at baseline (~23.0%) and increased inadequately over the study period to 193 (40.5%) in the < 40‐years group vs. 375 (34.9%) in the ≥ 40‐years group, respectively (not shown in Tables). Similarly, direct acting antivirals (DAA) receipt at baseline was only numerically lower in the < 40 years vs. the older group (10.6% vs. 17.9%, *p* = 0.2), reaching 26 (55.3%) and 198 (51.3%), respectively, over the study duration (not shown in Tables) (Table S2).

### 3.4 Patterns in the Incidence of aCLD From 2015 to 2023 (Table S3, Figures 1 and 2)

**FIGURE 1:**
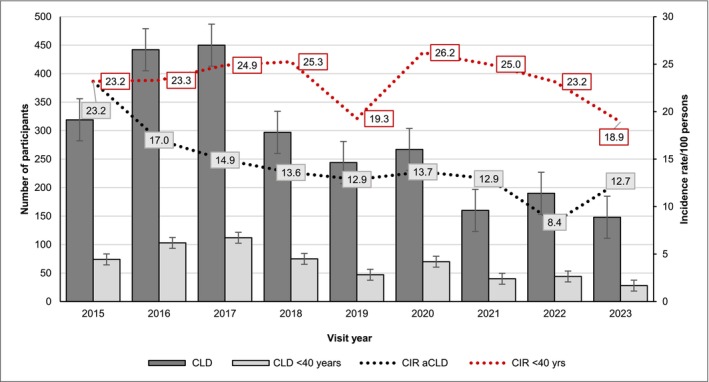
The crude incidence rate (CIR) of advanced chronic liver disease (aCLD) over study period. Persons with aCLD < 40 years of age at diagnosis are presented. ACLD = advanced chronic liver disease.

**FIGURE 2:**
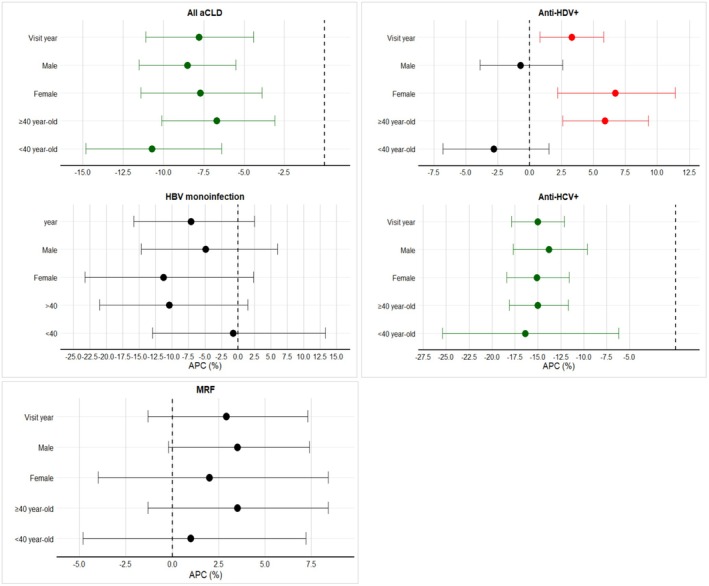
Annual percentage change (APC) of advanced chronic liver disease (aCLD) over study period (2015–2023) by sex and age group for all cohort, HBsAg+, HBV monoinfection, anti‐HDV+, anti‐HCV+ and MRF (metabolic risk factor). APC = annual percentage change; HBsAg+ = hepatitis B surface antigen; Anti‐HDV + = HBsAg+/anti‐HDV+ or HDV RNA+; HBV monoinfection = HBsAg+/anti‐HDV‐ and no record of HDV RNA+; Anti‐HCV + = anti‐hepatitis C virus antibody + or HCV RNA+; MRF = metabolic risk factor. Green dot means a decline in APC, red dot means an increase in APC, black dot means no change in APC; horizontal dotted line marks APC = 0.

The number of cases and the proportion of aCLD by sex, age group, viral hepatitis and MRF are shown in Table S3, the prevalence by visit year in Figure 1, and the APC is illustrated in Figure 2. Among all individuals, the incidence rates of aCLD decreased significantly from 2015 to 2023, showing APC = −7.8% (−11.1% to −4.4%; *p* < 0.001).

The incidence rates of HBsAg+ related aCLD increased (APC = 5.0%; 2.7% to 7.3%; *p* < 0.001), while anti‐HDV+ − related aCLD increased in female patients (APC = 6.7%; 2.2% to 11.4%; *p* = 0.003) and remained steady in male patients (APC = −0.7; −3.9% to 2.6%; *p* = 0.67).

By age groups, those aged < 40 years had a steady incidence for HBsAg+ (APC = −0.2%; −3.9% to 3.7%; *p* = 0.93), for anti‐HDV+ (APC = −2.8; −6.8% to 1.5%; *p* = 0.19) and a declining incidence for anti‐HCV+ (APC = −16.4%; −25.4% to −6.2%; *p* < 0.001). Whereas for those ≥ 40 years, an increase in incidence rates of aCLD occurred due to HBsAg+ (APC = 7.2%; 3.9% to 10.5%; *p* < 0.001), mostly attributed to anti‐HDV+ (APC = 5.9%; 2.6% to 9.3%; *p* < 0.001), while a decline was noted for anti‐HCV+ (APC = −15.0%; −18.1% to −11.7%; *p* < 0.001), but a steady incidence of MRF (APC = 3.5%; −1.3% to 8.4%; *p* = 0.15).

Overall persons with anti‐HCV+ showed a declining incidence rate of aCLD from 57.4% to 20.3% (APC = −15.0%; −17.9% to −12.1%; *p* < 0.001). Female patients experienced a greater decline than males (APC = −15.1%; −18.4% to −11.6%; *p* < 0.001). From 2015 to 2023, the prevalence of MRF was 30.1% and 39.2%, with an overall steady incidence of MRF‐related aCLD across the study period, with the APC showing a positive direction, however not statistically significant (APC = 2.9%; −1.3% to 7.3%; *p = 0.18*). (Table S3, Figure 2).

### 3.5 Parameters Associated With aCLD Diagnosis in All Participants

In univariable logistic regression analysis, variables that were significantly associated with aCLD were age older than 40, male sex, HBsAg+, HBeAg+, Anti‐HDV+, HDV RNA+, anti‐HCV+ and HCV RNA+. Other factors significantly (positively associated) were elevated ALT, AST, GGT and diabetes mellitus. Compared to low‐risk PAGE‐B, intermediate‐risk carried 4.12 odds (3.64–4.66) and high‐risk carried 16.14 odds (9.51–27.40) of aCLD. MRF had a negative association with aCLD in this cohort, showing 9% lower odds (0.83–0.99). In the multivariable model, variables independently associated with aCLD were age ≥ 40 years carrying 1.85 higher odds (1.58–2.17); male sex with 1.30 higher odds (1.13–1.49); HBsAg+ with 1.55 odds (1.18–2.03); anti‐HDV+ with 1.49 odds (1.15–1.93); HCV RNA+ with 1.71 odds (1.36–2.15); elevated ALT carrying 1.70 odds (1.41–2.02); elevated AST with 1.28 odds (1.10–1.48); elevated GGT at 1.43 odds (1.24–1.64); and diabetes mellitus carrying 1.80 odds (1.44–2.25). Meanwhile, MRF showed a negative association with 21% lower odds for aCLD (aOR = 0.79, 0.70–0.91). (Table 2).

**TABLE 2:** Association of baseline characteristics with advanced chronic liver disease (aCLD) in 17,711 participants in univariable and multivariable logistic regression models.

Parameters	Univariable		Multivariable	
	OR (95% CI)	*p*	aOR (95% CI)	*p*
Age ≥ 40 years‐old	2.36 (2.14–2.60)	< 0.001	1.85 (1.58–2.17)	< 0.001
Sex, male (female reference)	1.13 (1.04–1.23)	0.005	1.30 (1.13–1.49)	0.004
BMI, ≥ 23.0	1.12 (0.94–1.35)	0.21		
HBsAg+	1.60 (1.47–1.74)	< 0.001	1.55 (1.18–2.03)	0.002
HBeAg+	1.11 (0.43–2.88)	0.83		
Anti‐HDV+	2.15 (1.97–2.34)	< 0.001	1.49 (1.15–1.93)	0.003
HDV RNA+	2.02 (1.84–2.21)	< 0.001	1.20 (0.54–2.65)	0.65
Anti‐HCV+	0.67 (0.07–6.48)	0.73		
HCV RNA+	2.16 (1.53–3.04)	< 0.001	1.71 (1.36–2.15)	< 0.001
ALT, IU/L, above ULN	3.37 (3.04–3.75)	< 0.001	1.70 (1.41–2.02)	< 0.001
AST, IU/L, above ULN	2.30 (2.11–2.51)	< 0.001	1.28 (1.10–1.48)	0.001
GGT, IU/L, above ULN	3.61 (3.26–4.00)	< 0.001	1.43 (1.24–1.64)	< 0.001
Diabetes mellitus	2.07 (1.21–3.54)	0.008	1.80 (1.44–2.25)	< 0.001
PAGE‐B score^ (low risk reference)				
Intermediate	4.12 (3.64–4.66)	< 0.001		
High	16.14 (9.51–27.40)	< 0.001		
MRF	0.91 (0.83–0.99)	0.033	0.79 (0.70–0.91)	0.001

*Note:* ^Assessed in patients with HBsAg+.

Abbreviations: ALT = alanine aminotransferase, AST = aspartate aminotransferase, BMI = body mass index, FBG = fasting blood glucose, GGT = gamma glutamyl transferase, HBsAg = hepatitis B surface antigen, HCV = hepatitis C virus, HDV = hepatitis D virus, OR = odds ratio, aOR = adjusted odds ratio, ULN = upper limit of normal. Multivariable model adjusted for age, sex, HBsAg+, anti‐HDV+, HCV RNA+, ALT, AST, GGT, and FBG.

## 4 Discussion

This large‐scale analysis of 17,711 Mongols during 2015–2023 reveals that most advanced chronic liver diseases (aCLD) are attributed to chronic viral hepatitis B or B/D in males and chronic hepatitis C and B/D in females. Although the overall prevalence of aCLD declined over the study period, largely due to declining hepatitis C, the burden of hepatitis B and D showed increasing trends regardless of age and sex.

At initial presentation, 14% of patients exhibited advanced liver disease, with only 21% at low risk of HCC. Notably, only 25% of patients were receiving antiviral therapy at the time of referral/visit. Males with aCLD were diagnosed at a younger age, with 95% at increased risk of developing HCC within 5 years. The majority of patients with aCLD and anti‐HCV+ (*n* = 1004) had HCV RNA replication (*n* = 787, 78.4%) indicating delayed linkage to care. Diabetes mellitus remained a significant factor associated with aCLD in adjusted analyses.

This study provides a comprehensive epidemiological analysis of a symptomatic population with liver diseases in Mongolia, a Central‐Eastern Asian country heavily burdened by viral hepatitis B, C and D [14]. The findings underscore the need for enhanced screening of chronic liver disease, improved access to therapy and further research into other environmental and behavioural factors influencing liver disease progression in Mongolia.

Previous studies from Mongolia estimated *a* 70% prevalence of HBsAg+, 51.2% of HCV RNA+ and 56.5% of HDV RNA+ in patients with CLD [15], while lower estimates of 40% HBsAg+, 39% anti‐HCV+ and 20% dual infections were found in patients with cirrhosis [16].

In a 2006 study of 292 Mongol patients, HBV/HDV+ constituted 74.4% of chronic hepatitis diagnosed at a ~ mean age of 43 years, while it was 49% for HCV monoinfection [17]. This differs from the present analysis, where a higher prevalence of HBV/HDV+ at 78.6% and HCV RNA+ at 57.9% is likely due to the more advanced disease in our cohort. However, our results align with a more recent analysis attributing two‐thirds of liver cancer‐related mortality to HBV and HCV, reflecting the predominant aetiology for the long‐term risk in patients with aCLD [18].

Populations with HBV and HDV in Mongolia show different characteristics compared to Western‐based cohorts. For instance, a lower prevalence of HIV could be noted compared to studies from the United States (US) reporting 30.9% HIV prevalence in 6719 HBV/HDV coinfected patients [19] and 13.9% from another report [20]. Of note, the prevalence of aCLD in the present analysis aligns with US findings of 12.7%–31.7% cirrhosis in HBV/HDV cohorts but differs from the rather high prevalence of diabetes (20.0%–50.0%) compared to ours (13.5%). This negligible prevalence of HIV+ in the present cohort (only 1 patient of 1312 tested) reflects a difference in modes of transmission, where intravenous drug use and risky behaviour possibly dominate routes of transmission in Western settings. Nevertheless, we could demonstrate a positive trend of MRF over study years among persons with aCLD, with diabetes being significantly associated with aCLD, particularly in patients over 40 years of age, in agreement with prior analyses [21, 22].

In the present study, female patients with aCLD had a higher prevalence of anti‐HCV+ (66.8%), compared to males (47.4%), possibly due to exposure to obstetric procedures [14]. However, the detection of HCV RNA replication in 78% of patients with aCLD at diagnosis, more so in females, highlights significant gaps in linkage to care. Earlier studies in Mongolia reported a high prevalence of triple infections (HBV, HDV, HCV) at 63.2% in HCC compared to 14.4% in CLD [15]. A review of consecutive Mongol patients admitted to the National Centres for Cancer and Communicable Diseases from 2000 to 2009 revealed that in 941 patients with cirrhosis, 40% had HBsAg+, 39% had anti‐HCV+ and 20% had dual HBV/HCV coinfection, with more HCV infection among females and patients older than 45 years [16]. In our study, triple infection was observed in 6.4% of HBsAg+ patients supporting the decline in HCV prevalence and possibly reduced survival of patients with multiple infections. This, while our finding that only 25% and 17% of patients with aCLD were on NAs and DAAs, respectively, highlights a missed opportunity for linkage to care in the majority and suboptimal treatment coverage.

Effective and affordable oral therapies for HBV/HDV infection remain an unmet need. Recent consensus guidelines recommend considering all patients with HDV RNA replication for therapy, given the accelerated disease progression in HBV/HDV co‐infection [23]. While the newly approved hepatocyte entry inhibitor, bulevirtide, has shown promising results, its cost and limited availability make pegylated‐interferon the more feasible option in resource‐limited settings, despite its suboptimal virological response and recognised side effects [24]. Although there are allocated funds for the expense of medication for hepatitis D in the state budget starting from 2025 in Mongolia [25], access to bulevirtide treatment remains highly limited, considering the high number of infected persons.

Early screening and diagnosis of HBV/HDV before advanced disease could increase the number of patients eligible for interferon therapy, potentially improving liver disease outcomes [24]. Importantly, our findings that a large proportion of patients are at intermediate and high risk of HCC warrant vigilant national efforts to increase access to therapy and improve HCC surveillance.

While viral hepatitis (HBV, HDV, HCV) dominated aCLD aetiology in our cohort, the rising burden of metabolic dysfunction‐associated steatotic liver disease (MASLD) in Asian countries, possibly driven by urbanisation and increasing obesity and diabetes prevalence, may influence future aCLD trends [6]. In our analysis, metabolic risk factors (MRF, including BMI ≥ 23.0 kg/m^2^ or diabetes, present in 37.7% of aCLD cases) showed a steady prevalence trend (APC 2.9%, 95% CI, −1.3%–7.3%, *p* = 0.18) but a negative association with aCLD in multivariable analysis (aOR = 0.79, 95% CI, 0.70–0.91). This may reflect underdiagnosis of MASLD due to the overriding focus on viral hepatitis in clinical settings or less advanced MASLD at presentation, as viral etiologies likely accelerate fibrosis progression. The steady MRF trend, contrasted with declining HCV (APC −15.0%) and rising HDV (APC 3.3%), suggests a growing role of MASLD, warranting enhanced screening for metabolic factors alongside viral hepatitis in Mongolia.

The present study provides a detailed clinical and epidemiological characterisation of aCLD rather than summary measures from a hyperendemic region with a sample size of ~18,000 individuals possibly representative of populations in hepatology clinics in Central and Eastern Asia. Large scale studies are lacking from Central Asia despite the heavy burden of liver disease in this region [1]. Our findings could be applicable to similar low and middle‐income Asian urban settings with endemic HBV/HDV infection, offering evidence‐based insights to guide policies for resource allocation. We assume a homogeneous level of service at the liver centre, minimising ascertainment bias related to variations in virological test performance across laboratories.

Nevertheless, we acknowledge some limitations in the current study. The cross‐sectional design of the study limits causal inference and addressing rates of disease progression and treatment outcomes. The hepatology‐centre based cohort might be enriched for more advanced cases. Therefore, our findings might overestimate aCLD due to this selection bias limiting general population representativeness. We lack data on alcohol overconsumption, which is a significant risk factor for aCLD. Differences in alcohol consumption might partially explain the more frequently observed presence of aCLD among men found in our study [18]. Detailed granular information on the routes of transmission, sociodemographic factors, comorbidities, therapies such as statins or aspirin, as well as barriers to antiviral treatment uptake were not captured in the database, all of which might influence care‐seeking, adherence and liver disease progression. Longitudinal studies adjusting for these factors are warranted. Nevertheless, future studies addressing the potential barriers to treatment uptake, access to novel therapies, and logistical challenges in Mongolia are critical to better design linkage to care strategies.

In conclusion, the burden of liver disease due to chronic viral hepatitis B and D remains high in Mongolia with a minority of patients receiving antiviral therapy against these infections, while a decline in hepatitis C is observed. Middle‐aged individuals are affected the most, especially male individuals, highlighting the need for early diagnosis and treatment to halt liver disease progression. Continuation of the ongoing national initiatives promoting awareness of hepatocellular carcinoma, enhancing surveillance and ensuring widespread access to new antivirals against hepatitis B/D are needed to reduce the burden of liver cancer in Mongolia.

## Author Contributions

**Habiba Kamal:** conception, analysis and writing of the manuscript. **Ganbolor Jargalsaikhan**, and **Sanjaasuren Enkhtaivan:** resources, review and editing. **Daniel Bruce:** analysis, review and editing. **Karin Lindahl:** review and editing. **Tuvshinjargal Ulziibadrakh**, **Munguntsetseg Batkhuu**, **Purevjargal Bat‐Ulzii**, and **Sumiya Byambabaatar:** resources, review and editing. **Naranjargal B Dashdorj:** resources, senior supervision, review and editing. **Soo Aleman:** conception, senior supervision, review and editing. All authors approved the final version.

## Funding

The authors have nothing to report.

## Conflicts of Interest

The authors declare no conflicts of interest.

## Supporting information


**Data S1:** Table S1: Baseline characteristics of patients with advanced chronic liver disease (aCLD) subgrouped by sex.
**Table S2:** Baseline characteristics of patients with advanced chronic liver disease (aCLD) subgrouped by age 40 years.
**Table S3:** Incidence* (number of cases divided by total attendees per respective year) and annual percentage change (APC) of advanced chronic liver disease (aCLD) from 2015 to 2023, by subgroups.
**Figure S1:** Study flowchart.
**Figure S2:** Proportions of persons with record of HBsAg+, anti‐HDV+, anti‐HCV+, no viral hepatitis B, C or D and metabolic risk factors (MRF) among advanced chronic liver disease (aCLD) over study period.
**Figure S3:** PAGE‐B score categories to predict 5‐year risk of hepatocellular carcinoma among patients with advanced chronic liver disease (aCLD) and chronic hepatitis B sub grouped by sex and age 40 years at diagnosis.

## Data Availability

The data that support the findings of this study are available on request from the corresponding author. The data are not publicly available due to privacy or ethical restrictions.
