# Supplementary material for: Trends of Advanced Chronic Liver Disease Among 17,711 Persons in Mongolia During Years 2015–2023
Source: J Viral Hepat. 2026 Jan 6;33(2):e70129. doi: 10.1111/jvh.70129 (PMC12771268; doi:10.1111/jvh.70129)
Supplement: Supplementary file 1 — Data S1: Table S1: Baseline characteristics of patients with advanced chronic liver disease (aCLD) subgrouped by sex. Table S2: Baseline characteristics of patients with advanced chronic liver disease (aCLD) subgrouped by age 40 years. Table S3: Incidence* (number of cases divided by total attendees per respective year) and annual percentage change (APC) of advanced chronic liver disease (aCLD) from 2015 to 2023, by subgroups. Figure S1: Study flowchart. Figure S2: Proportions of persons with record of HBsAg+, anti‐HDV+, anti‐HCV+, no viral hepatitis B, C or D and metabolic risk factors (MRF) among advanced chronic liver disease (aCLD) over study period. Figure S3: PAGE‐B score categories to predict 5‐year risk of hepatocellular carcinoma among patients with advanced chronic liver disease (aCLD) and chronic hepatitis B sub grouped by sex and age 40 years at diagnosis. [file JVH-33-0-s001.docx]

**Trends of advanced chronic liver disease among 17,711 persons** **in Mongolia during years 2015-2023**

Habiba Kamal^1,2^ , Ganbolor Jargalsaikhan^3,4^, Sanjaasuren Enkhtaivan^3,4^, Daniel Bruce^5^, Karin Lindahl^1,2^, Bekhbold Dashtseren^3,4^, Tuvshinjargal Ulziibadrakh^3,4^, Munguntsetseg Batkhuu^3,4^, Purevjargal Bat-Ulzii^3,4^, Sumiya Byambabaatar^3,4^, Soo Aleman^#1,2^ and Naranjargal B Dashdorj^#3,4^

#= shared last authorship.

Affiliations

1. Department of Medicine Huddinge, Karolinska Institute, Stockholm, Sweden
2. The department of Infectious Diseases, Karolinska University Hospital, Stockholm, Sweden
3. The Liver Center, Ulaanbaatar, Mongolia
4. ONOM Foundation, Ulaanbaatar, Mongolia

5. Cytel Statistical Consultancy, Stockholm, Sweden

| **sTable 1: Baseline characteristics of patients with advanced chronic liver disease (aCLD) subgrouped by sex** | | | | | |
| --- | --- | --- | --- | --- | --- |
| **Variables** | **Number** | **All** | **Female** | **Male** | **p-value** |
| Number |  | 2,517 (100) | 1,338 (53.2) | 1,179 (46.8) | 0.005 |
| Age at HBsAg test, years, mean (sd) | 2,517 | 50.1 (12.0) | 53.6 (11.2) | 46.1 (11.6) | <0.001 |
| Age below 40 years of age | 2,517 | 593 (23.6) | 189 (14.1) | 404 (34.3) | <0.001 |
| BMI, Kg/m^2^, median (IQR) | 792 | 26.7 (23.9, 30.7) | 26.3 (23.6, 30.1) | 27.2 (24.2, 30.9) | 0.14 |
| ALT, IU/L, median (IQR) | 2,442 | 68.6 (39.9, 117.0) | 59.0 (34.6, 98.4) | 82.6 (48.2, 136.5) | <0.001 |
| ALT level category | 2,442 |  |  |  |  |
| Normal |  | 482 (19.7) | 265 (20.2) | 217 (19.2) | 0.13 |
| Elevated >*ULN |  | 1,960 (80.3) | 1,049 (79.8) | 911 (80.8) | 0.61 |
| AST, IU/L, median (IQR) | 2,440 | 62.0 (38.6, 98.2) | 60.7 (38.3, 93.2) | 63.6 (39.0, 103.7) | 0.055 |
| GGT, IU/L, median (IQR) | 1,888 | 63.9 (35.2, 119.9) | 51.4 (28.9, 93.3) | 82.4 (44.8, 152.8) | <0.001 |
| Elevated GGT above ULN | 1,888 | 1,243 (65.8) | 646 (64.2) | 597 (67.8) | 0.10 |
| Platelets count, 10*9 cells/L , median (IQR) | 2,437 | 123.0 (94.0, 142.0) | 119.0 (91.0, 140.0) | 127.0 (97.0, 145.0) | <0.001 |
| FBG level, mmol/L, median (IQR) | 2,051 | 5.1 (4.6, 5.8) | 5.0 (4.5, 5.6) | 5.2 (4.8, 5.9) | <0.001 |
| Diabetes mellitus | 2,051 | 277 (13.5) | 122 (11.2) | 155 (16.2) | <0.001 |
| **Virological Parameters** |  |  |  |  |  |
| No record of viral hepatitis B, C, D | 2,517 | 148 (5.9) | 59 (4.4) | 89 (7.5) | 0.001 |
| HBsAg+ | 2,517 | 1,550 (61.6) | 746 (55.8) | 804 (68.2) | <0.001 |
| HBeAg positive | 308 | 135 (43.8) | 51 (34.2) | 84 (52.8) | 0.001 |
| ***Any anti-HDV or HDV RNA test record*** | **2,517** | **1,578 (62.7)** | **803 (60.0)** | **775 (65.7)** | **0.003** |
| HBsAg+/anti-HDV- | 2,298 | 90 (5.7) | 32 (4.0) | 58 (7.5) | 0.004 |
| Anti-HDV+ | 1,578 | 1,241 (78.6) | 598 (74.5) | 643 (83.0) | <0.001 |
| HDV RNA+ | 1,241 | 802 (64.6) | 408 (68.2) | 394 (61.3) | 0.012 |
| ***Any anti-HCV or HCV RNA test record*** | **2,517** | **1,733 (68.9)** | **939 (70.2)** | **794 (67.3)** | **0.13** |
| Anti-HCV+ or HCV RNA+ | 1,733 | 1,004 (57.9) | 627 (66.8) | 377 (47.4) | <0.001 |
| HCV RNA+# | 1,004 | 787 (78.4) | 505 (80.5) | 282 (74.8) | <0.001 |
| LSM, kPa, median (IQR) | 702 | 17.6 (14.4, 24.8) | 17.0 (14.2, 23.9) | 18.4 (14.5, 25.9) | 0.019 |
| Previous NAs | 1,550 | 388 (25.0) | 177 (23.7) | 182 (22.6) | 0.60 |
| Previous DAAs | 433 | 74 (17.1) | 52 (18.3) | 22 (14.8) | 0.41 |
| MRF | 2,517 | 949 (37.7) | 461 (34.5) | 488 (41.4) | <0.001 |
| Decompensation | 2,517 | 127 (5.0) | 75 (5.6) | 52 (4.4) | 0.52 |
| HCC | 2,517 | 46 (1.8) | 20 (1.5) | 26 (2.2) | 0.18 |
| *Parameters are provided as n (%) unless stated otherwise. Abbreviations: IQR=interquartile range; BMI=body mass index; BMI≥23.0 is cutoff for overweight/obese in Asian population; ALT=alanine aminotransferase; AST=aspartate aminotransferase; GGT=gamma glutamyl transferase; AFP=alpha-fetoprotein; FBG=fasting blood glucose; LSM=liver stiffness measurement; NAs= nucleotide analogues; DAAs= direct anti-viral agents; MRF=cardiometabolic risk factor; HCC= hepatocellular carcinoma.# of those tested for HCV RNA.* | | | | | |

| **sTable 2: Baseline characteristics of patients with advanced chronic liver disease (aCLD) subgrouped by age 40 years** | | | | | |
| --- | --- | --- | --- | --- | --- |
| **Variables** | **N** | **All** | **<40 years** | **≥40 years** | **p-value** |
| **number** |  | **2,517** | **593** | **1,924** |  |
| Age at HBsAg test, years, mean (sd) | 2,517 | 50.8 (40.7, 58.7) | 34.9 (31.6, 37.6) | 54.4 (47.7, 61.0) | <0.001 |
| Female | 2,517 | 1,338 (53.2) | 189 (31.9) | 1,149 (59.7) | <0.001 |
| Male, |  | 1,179 (46.8) | 404 (68.1) | 775 (40.3) |  |
| BMI, Kg/m^2^, median (IQR) | 792 | 26.7 (23.9, 30.7) | 26.2 (22.6, 30.8) | 26.8 (24.2, 30.5) | 0.040 |
| BMI ≥23.0 | 792 | 621 (78.4) | 143 (68.4) | 478 (82.0) | <0.001 |
| ALT, IU/L, median (IQR) | 2,442 | 68.6 (39.9, 117.0) | 69.5 (42.6, 119.3) | 68.1 (39.0, 116.3) | 0.12 |
| ALT level category | 2,442 |  |  |  |  |
| Normal |  | 482 (19.7) | 111 (19.5) | 371 (19.8) | 0.08 |
| Elevated* >ULN |  | 1,960 (80.3) | 459 (80.5) | 1,501 (80.2) | 0.91 |
| AST, IU/L, median (IQR) | 2,440 | 62.0 (38.6, 98.2) | 54.1 (34.5, 91.6) | 63.8 (40.0, 99.6) | <0.001 |
| GGT, IU/L, median (IQR) | 1,888 | 63.9 (35.2, 119.9) | 64.0 (35.9, 132.4) | 63.9 (35.0, 118.5) | 0.51 |
| Elevated GGT above ULN | 1,888 | 1,243 (65.8) | 290 (62.6) | 953 (66.9) | 0.094 |
| Platelets count, 10*9 cells/L, median (IQR) | 2,437 | 123.0 (94.0, 142.0) | 131.0 (97.0, 147.0) | 121.0 (93.0, 141.0) | <0.001 |
| FBG level, mmol/L, median (IQR) | 2,051 | 5.1 (4.6, 5.8) | 4.9 (4.5, 5.5) | 5.1 (4.7, 5.9) | <0.001 |
| Diabetes mellitus | 2,051 | 277 (13.5) | 46 (9.7) | 231 (14.6) | 0.006 |
| AFP, ng/mL, median (IQR) | 1,685 | 8.0 (4.1, 19.6) | 5.6 (3.2, 12.4) | 8.7 (4.4, 21.5) | <0.001 |
| **Virological Parameters** |  |  |  |  |  |
| HBsAg+ | 2,517 | 1,550 (61.6) | 477 (80.4) | 1,073 (55.8) | <0.001 |
| HBeAg positive | 308 | 135 (43.8) | 55 (47.8) | 80 (41.5) | 0.30 |
| ***Any anti-HDV or HDV RNA test record*** | **2,517** | **1,578 (62.7)** | **440 (74.2)** | **1,138 (59.1)** | **<0.001** |
| HBV monoinfection | 2,298 | 105 (4.2) | 40 (6.7) | 65 (3.4) | <0.001 |
| Anti-HDV+ or HDV RNA+ | 1,578 | 1,241 (78.6) | 389 (88.4) | 852 (74.9) | <0.001 |
| HDV RNA+ | 1,241 | 802 (64.6) | 258 (66.3) | 544 (63.8) | <0.001 |
| ***Any anti-HCV or HCV RNA test record*** | **2,517** | **1,733 (68.9)** | **343 (57.8)** | **1,390 (72.2)** | **<0.001** |
| Anti-HCV+ | 1,733 | 1,004 (57.9) | 118 (34.4) | 886 (63.7) | <0.001 |
| HCV RNA+ | 1,004 | 787 (78.4) | 77 (65.3) | 710 (80.1) | <0.001 |
| No record of viral hepatitis B, C, D | 2,517 | 148 (5.9) | 39 (6.6) | 109 (5.7) | 0.40 |
| LSM, kPa, median (IQR) | 702 | 17.6 (14.4, 24.8) | 17.3 (14.3, 23.9) | 18.1 (14.4, 25.1) | 0.12 |
| Previous NAs | 1,550 | 388 (25.0) | 111 (23.3) | 248 (23.1) | 0.91 |
| Previous DAAs | 433 | 74 (17.1) | 5 (10.6) | 69 (17.9) | 0.20 |
| MRF | 2,517 | 949 (37.7) | 194 (32.7) | 755 (39.2) | 0.004 |
| HCC | 2,517 | 46 (1.8) | 1 (0.2) | 45 (2.3) | <0.001 |
| *Parameters are provided as n (%) unless stated otherwise. Abbreviations: IQR=interquartile range; BMI=body mass index; BMI≥23.0 is cutoff for overweight/obese in Asian population; ALT=alanine aminotransferase; AST=aspartate aminotransferase; GGT=gamma glutamyl transferase; AFP=alpha-fetoprotein; FBG=fasting blood glucose; LSM=liver stiffness measurement; NAs= nucleotide analogues; DAAs= direct acting anti-viral; MRF=metabolic risk factor; HCC= hepatocellular carcinoma.# of those tested for HCV RNA.* | | | | | |

| **sTable 3: Incidence* (number of cases divided by total attendees per respective year) and annual percentage change (APC) of advanced chronic liver disease (aCLD) from 2015 to 2023, by subgroups** | | | | | | |
| --- | --- | --- | --- | --- | --- | --- |
| Group | Number (IR%) 2015 | Number (IR%) 2023 | APC | LCI | UCI | p-value |
| **All** |  |  |  |  |  |  |
| Visit year | **319 (23.2)** | **148 (12.7)** | **-7.8** | **-11.1** | **-4.4** | <0.001 |
| Female | 194 (22.5) | 75 (12.6) | **-7.7** | **-11.4** | **-3.9** | <0.001 |
| Male | 125 (24.2) | 73 (12.8) | **-8.5** | **-11.5** | **-5.5** | <0.001 |
| <40-year-old | 74 (14.7) | 28 (6.2) | **-10.7** | **-14.8** | **-6.4** | <0.001 |
| ≥40-year-old | 245 (28.0) | 120 (16.8) | **-6.7** | **-10.1** | **-3.1** | <0.001 |
| **HBsAg+** |  |  |  |  |  |  |
| Visit year | 189 (59.2) | 111 (75.0) | **5.0** | **2.7** | **7.3** | <0.001 |
| Female | 98 (50.5) | 55 (73.3) | **8.2** | **4.2** | **12.4** | <0.001 |
| Male | 91 (72.8) | 56 (76.7) | **1.4** | **-1.5** | **4.3** | 0.35 |
| <40-year-old | 61 (82.4) | 22 (78.6) | **-0.2** | **-3.9** | **3.7** | 0.93 |
| ≥40-year-old | 128 (52.2) | 89 (74.2) | **7.2** | **3.9** | **10.5** | <0.001 |
| **Anti-HDV+** |  |  |  |  |  |  |
| Visit year | 130 (40.8) | 89 (60.1) | **3.3** | **0.8** | **5.8** | 0.01 |
| Female | 66 (34.0) | 44 (58.7) | **6.7** | **2.2** | **11.4** | 0.003 |
| Male | 64 (51.2) | 45 (61.6) | -0.7 | -3.9 | 2.6 | 0.67 |
| <40-year-old | 40 (54.1) | 16 (57.1) | -2.8 | -6.8 | 1.5 | 0.19 |
| ≥40-year-old | 90 (36.7) | 73 (60.8) | **5.9** | **2.6** | **9.3** | <0.001 |
| **Anti-HCV+** |  |  |  |  |  |  |
| Visit year | 183 (57.4) | 30 (20.3) | **-15.0** | **-17.9** | **-12.1** | <0.001 |
| Female | 123 (63.4) | 19 (25.3) | **-15.1** | **-18.4** | **-11.6** | <0.001 |
| Male | 60 (48.0) | 11 (15.1) | **-13.8** | **-17.7** | **-9.6** | <0.001 |
| <40-year-old | 22 (29.7) | 3 (10.7) | **-16.4** | **-25.4** | **-6.2** | <0.001 |
| ≥40-year-old | 161 (65.7) | 27 (22.5) | **-15.0** | **-18.1** | **-11.7** | <0.001 |
| **MRF** |  |  |  |  |  |  |
| Visit year | 96 (30.1) | 58 (39.2) | 2.9 | -1.3 | 7.3 | 0.18 |
| Female | 50 (25.8) | 25 (33.3) | 2.0 | -4.0 | 8.4 | 0.52 |
| Male | 46 (36.8) | 33 (45.2) | 3.5 | -0.2 | 7.4 | 0.07 |
| <40-year-old | 22 (29.7) | 11 (39.3) | 1.0 | -4.8 | 7.2 | 0.74 |
| ≥40-year-old | 74 (30.2) | 47 (39.2) | 3.5 | -1.3 | 8.4 | 0.15 |
| *Only cases with viral hepatitis markers were considered. Abbreviations; LCI=lower confidence interval; UCI=upper confidence interval; MRF=metabolic risk factor. | | | | | | |

**sFigure 1: Study flowchart**

Attendees with available HBsAg test

(n=51,113)

<18 years of age, missing data on birth date and sex

(n=2,591)

Adults with HBsAg test

(n=48,522)

No available LSM or platelet count (n=30,811)

Persons with available LSM and/or platelet test

(n=17,711)

**Advanced CLD**

**(n=2,517)**

No aCLD

(n=15,194)

Abbreviations:

HBsAg: hepatitis B surface antigen; HCC=hepatocellular carcinoma; LSM= liver stiffness measurement; Advanced chronic liver disease (CLD) defined as LSM≥12.5 and/or platelets count <150*10^9^ cells/L

sFigure 2: Proportions of persons with record of HBsAg+, anti-HDV+, anti-HCV+, no viral hepatitis B, C or D and metabolic risk factors (MRF) among advanced chronic liver disease (aCLD) over study period

Patients with missing test were considered true negative and some patients might fit into different categories. Abbreviations: HBsAg+=hepatitis B surface antigen; HDV= hepatitis D virus; HCV=hepatitis C virus; MRF=metabolic risk factor.

sFigure 3: PAGE-B score categories to predict 5-year risk of hepatocellular carcinoma among patients with advanced chronic liver disease (aCLD) and chronic hepatitis B subgrouped by sex and age 40 years at diagnosis.

Abbreviations: PAGE-B score=platelets age gender risk score; patients with high, intermediate, and low PAGE-B score had 5-year cumulative hepatocellular carcinoma incidence rates of 17%, 3%, and 0%. respectively (13).
